# Teaching Health Workers Malaria Diagnosis

**DOI:** 10.1371/journal.pmed.0020011

**Published:** 2005-02-22

**Authors:** Graham Icke, Richard Davis, William McConnell

## Abstract

In most parts of the world, microscopy is still the gold standard for diagnosing malaria. An online tool could help to improve your diagnostic skills

## Teaser

An online tool allows microscopists to test their knowledge

Malaria kills over one million people in Africa each year and contributes 10% of the continent's burden of disease [1]. One of the factors that affects the morbidity and mortality rate is incorrect diagnosis [2]. In this article, we describe a freely available online training tool for health professionals to learn malaria diagnosis. We describe why we launched it and discuss how it is being used.

## The Burden of Disease

Malaria affects at least 200–300 million people every year and causes from 1–2 million deaths—mostly children under five and pregnant women in sub-Saharan Africa [1]. These deaths largely occur in remote rural areas with poor access to health services. In non-pregnant adults, although mortality rates are lower, the debilitating disease affects quality of life. The economic burden is also extremely high, accounting for a reduction of 1.3% in the annual economic growth rate of countries where malaria is endemic [3]. Malaria costs Africa more than US$12 billion every year in lost GDP, even though it could be controlled for a fraction of that sum [1].

Malaria is not limited to Africa—40% of the world's population is at risk of acquiring the disease [4,5]—and it is crucial for all physicians, medical scientists, and other healthcare professionals to be alert to the diagnosis. In addition, each year, 25–30 million people from non-tropical countries visit areas where malaria is endemic [6], and between 10,000 and 30,000 contract malaria [7]. Some 90% of infected travellers do not become ill until they return home; this “imported malaria” is easily treated, but only if it is diagnosed promptly [2,8].

## The Importance of Correct Diagnosis

Despite the efforts of a global campaign to roll back the disease, the number of deaths from malaria is increasing in Africa [9]. This statistic highlights the importance of local capacity to diagnose and treat malaria in order to prevent illness and death [10]. In many parts of the world, education about malaria diagnosis and treatment is limited—and the incorrect diagnosis of malaria by clinical, laboratory staff, and other healthcare workers can contribute to morbidity and mortality [2,11].

There is another important reason why correct diagnosis matters. Because of rising drug resistance in Africa, the conventional drugs for treating malaria—chloroquine and sulfadoxine-pyrimethamine—are now failing in up to 80% of cases [12]. There is a highly effective alternative to these drugs, which is artemisinin-based combination therapy (ACT) [13], but ACT is expensive (an adult dose of chloroquine costs around 10 cents, whereas a dose of ACT costs at least ten times that amount) [14]. Scaling up the use of ACT is now a core strategy in the global campaign to control malaria. But in order to control costs, and to prevent the emergence of resistance to ACT, its use should be targeted to real (rather than presumptive) cases of malaria [14]. Currently, however, a comparison of the number of parasitologically confirmed cases of malaria with those that are presumptively diagnosed shows high rates of overdiagnosis outside of hospitals at the community level, where self treatment is routine [14].

## Microscopy as the Gold Standard for Diagnosis

**Figure 1:**
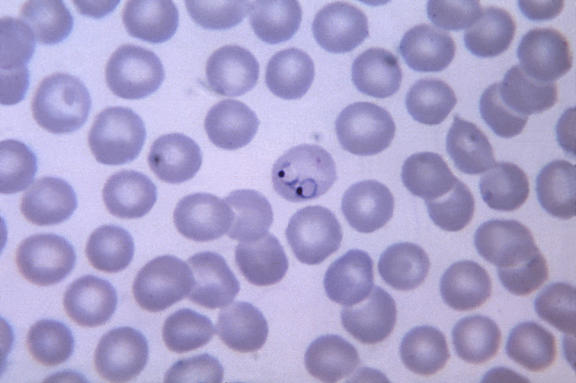
Thin Film Micrograph Showing a Red Blood Cell Containing Two Ring-Form P. vivax Parasites P. vivax rings have a large quantity of cytoplasm and a large chromatin dot, as well as occasional pseudopods. The red blood cells are normal, to 1.5× normal, sized, round, and contain fine Schüffner's dots, and they quite often contain multiple parasites. (Photograph: CDC/Dr Mae Melvin)

We still regard microscopy as the gold standard for the diagnosis and characterisation of malaria infection (Figure 1). Modern “dipstick” technology and molecular techniques have emerged as an aid to diagnosis [13,14]. Some of these are useful particularly for Plasmodium falciparum [15]—although they are less effective for other species or for mixed infections [16,17]—but these techniques are often only available in larger hospitals, which are more likely to be able to afford them.

Some authors have suggested that these new tests should not be considered a complete substitute for direct microscopic examination of blood smears [16]. We agree. While microscopy has limitations—morphology can be misleading if a patient has received partial treatment or incomplete prophylaxis, and in some locations a microscope (let alone a microscopist) can be rare—the new dipstick tests also have their flaws. For example, they must be stored correctly, used correctly, and interpreted correctly. There are concerns about the variability in the different tests' false positive and false negative rates and about their cost–benefit ratio. There are many supporters of the new dipstick technology, and as it improves and becomes more robust and reliable, it may well replace the microscope in practice. But for much of the world, the front-line diagnosis of malaria still remains in the hands of the reasonably trained microscopist.

Analysis of DNA by the polymerase chain reaction (PCR) may be a useful tool for diagnosis of malaria when the results of conventional techniques are negative, especially since PCR allows accurate species identification [18]. And when compared with the “gold standard” of microscopy, PCR has a sensitivity and specificity of 100%, with a detection limit of just one P. falciparum or three P. vivax parasites per microlitre of blood [19]. However, in most areas with malaria transmission, limited financial resources, persistent subclinical parasitaemia, and inadequate laboratory infrastructure preclude PCR as a routine diagnostic method [20]. Even in affluent, nonendemic countries, PCR is not a suitable method for routine use. Capital investment and ongoing running costs are prohibitive for many laboratories, and certainly for the foreseeable future this technology will remain a tool for the more specialised services in the more affluent societies. At this stage, it is not considered a “routine” assay.

## Teaching Microscopy Online

**Figure 2:**
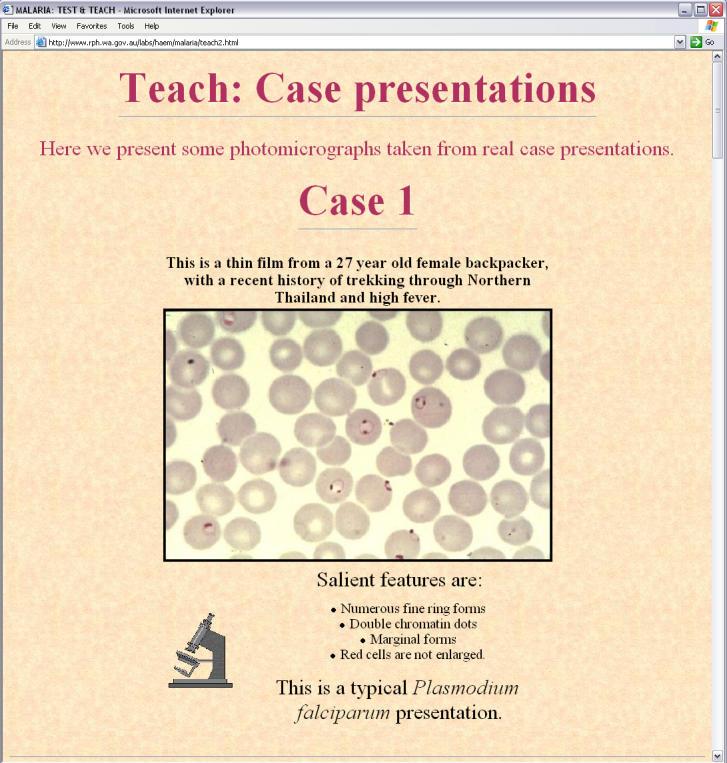
A Clinical Case from the “Test and Teach” Section

In the hope of improving health professionals' understanding of malaria diagnosis, treatment, and prophylaxis, we launched an online malaria education tool that went live in July 1998 (http://www.rph.wa.gov.au/labs/haem/malaria/index.html). The materials are now available in English, French, and Spanish. One of the most important features is a “Test and Teach” section to allow microscopists to develop and sharpen their skills in malaria diagnosis (Figure 2).

Because of regional variability in Internet access, we also made the tool freely available as a CD-ROM. The first CD-ROMs we produced, which were in English, reached institutions and centres in 41 countries during the first few months after the launch. By October 1999, we produced a new CD-ROM that incorporated a French translation of key areas of the project; a Spanish version followed. By the end of 1999, the CD-ROM version had reached over 70 countries and the website had received nearly 30,000 visitors.

To date, the website has received more than 500,000 visitors. Around 83 million pages of text were downloaded from the website between July 2001 and September 2003. The CD-ROM version (over 8,000 copies) has been sent on request to institutions and centres in 149 countries, a number that does not include unauthorised (pirated) copies of the CD-ROM. The number of hits to the site (the number of pages or images of the site that are accessed) has risen dramatically, from 1,500 to over 100,000 per week.

## Feedback from Users

Through a circulated questionnaire with a 63% response rate, we have received feedback from users in 15 countries. Respondents said they valued the content, presentation, and usefulness of the online information. Who is using the material? A wide range of people—from students through to experienced personnel, and from clinicians to scientific, research, and technical staff. Many tropical medicine institutions have requested copies of the CD-ROM to supplement their own training programs. We have discussed the project at scientific conferences around the world and in scientific journals, advertising the fact that this educational material is freely available. Institutions wishing to obtain a free copy of the CD-ROM should contact E-mail: Graham.Icke@health.wa.gov.au.

We also became aware that unauthorised copies of the CD-ROM were being produced. Although there is a real need to guarantee the integrity of the information on the CD-ROMs and ensure proper credit for the authors and patrons, our main aim is to spread the “good word”, and such unauthorised copies are a powerful aid to distribution. Given that there is no need to recoup profit from this project, our only concern is its usefulness. Consequently, we have welcomed the production of these pirated copies, on condition that they were of good quality and were made freely available at no cost.

## Conclusion

The Internet and associated technologies make it possible for educators with a desire to teach to contact those with a need to learn, regardless of the geographical distances involved. By enlisting assistance from international colleagues, language barriers can be overcome. In addition to the English, French, and Spanish versions, we are aware that our project has been translated into German, Thai, and Vietnamese and has been distributed through small regional health group networks.

There can be little doubt that these new technologies have the potential to revolutionise information dissemination, with particularly significant implications for healthcare professionals in developing countries. The malaria educational program could be a model on which future health education programs are based. With rapidly increasing access to these new information technologies, health care professionals from anywhere in the world can now join the global health community.

## Abbreviations

ACT: artemisinin-based combination therapy
PCR: polymerase chain reaction
